# Exercise rescues obese mothers’ insulin sensitivity, placental hypoxia and male offspring insulin sensitivity

**DOI:** 10.1038/srep44650

**Published:** 2017-03-14

**Authors:** Denise S. Fernandez-Twinn, Geraldine Gascoin, Barbara Musial, Sarah Carr, Daniella Duque-Guimaraes, Heather L. Blackmore, Maria Z. Alfaradhi, Elena Loche, Amanda N. Sferruzzi-Perri, Abigail L. Fowden, Susan E. Ozanne

**Affiliations:** 1 University of Cambridge Metabolic Research Laboratories and MRC Metabolic Diseases Unit, Wellcome Trust-MRC Institute of Metabolic Science, Cambridge, UK; 2 Department of Neonatal Medicine, University Hospital of Angers, Angers, France; 3 Department of Physiology, Development & Neuroscience, University of Cambridge, UK

## Abstract

The prevalence of obesity during pregnancy continues to increase at alarming rates. This is concerning as in addition to immediate impacts on maternal wellbeing, obesity during pregnancy has detrimental effects on the long-term health of the offspring through non-genetic mechanisms. A major knowledge gap limiting our capacity to develop intervention strategies is the lack of understanding of the factors in the obese mother that mediate these epigenetic effects on the offspring. We used a mouse model of maternal-diet induced obesity to define predictive correlations between maternal factors and offspring insulin resistance. Maternal hyperinsulinemia (independent of maternal body weight and composition) strongly associated with offspring insulin resistance. To test causality, we implemented an exercise intervention that improved maternal insulin sensitivity without changing maternal body weight or composition. This maternal intervention prevented excess placental lipid deposition and hypoxia (independent of sex) and insulin resistance in male offspring. We conclude that hyperinsulinemia is a key programming factor and therefore an important interventional target during obese pregnancy, and propose moderate exercise as a promising strategy to improve metabolic outcome in both the obese mother and her offspring.

Obesity is an epidemic affecting more than 1.4 billion adults worldwide. In 2014, over 300 million women were reported as obese (WHO Obesity and overweight Fact sheet No: 311, updated August 2014). Recent evidence suggests that 50% of women of reproductive age and 20–25% of pregnant women in Europe are overweight or obese at their first antenatal visit^1^. Obesity in women of childbearing age is particularly concerning as there is overwhelming evidence that being born to an obese mother not only has immediate detrimental effects on the mother and neonate, but also increases the risk for the child of developing metabolic disorders in later life^2^. Although some of this can be explained by transmission of obesity-susceptibility genes from mother to child, strong evidence from studies in humans^3^,^4^,^5^ and animal models^6^ suggests that epigenetic mechanisms programmed by *in utero* development in an obesogenic environment play an important role. For example, bariatric surgery to reduce weight in obese women has been shown to reduce the prevalence of obesity^3^,^4^,^5^, insulin resistance^4^,^5^ and raised blood pressure^5^ in the children born after surgery compared to their siblings born before surgery. These studies very clearly revealed that there is a disproportionate risk of disease in offspring born to the same mother and now living in the same current postnatal environment, but who were exposed to different *in utero* conditions. It highlights the awareness that optimizing the fetal environment is important if we are to prevent the epigenetic transmission of obesity from mother to child and to halt the obesity epidemic.

An important limitation in our ability to design intervention strategies to prevent the detrimental effects of maternal obesity on offspring health is that it is currently unclear which of the many potential variables associated with obesity is the key programming factor mediating the effects on the offspring. There are a number of potential candidates including hyperinsulinemia, hyperglycemia and hyperlipidemia^6^. Some of these factors (such as glucose) can move freely across the placenta, whilst others (such as insulin) cannot cross the placenta, thus would mediate their effects through actions on the placenta. Maternal obesity during pregnancy results in increased placental lipid accretion^7^,^8^ and also leads to a lipotoxic placental environment that is associated with increased markers of inflammation and oxidative stress and decreased regulators of angiogenesis^9^,^10^,^11^. Although there are a number of on-going randomized intervention studies in humans targeting the different potential programming factors associated with maternal obesity, including dietary, pharmacological and exercise intervention^12^,^13^,^14^, it is going to be many years before information is gained on the long term effects of the maternal intervention on the offspring.

Animal models are excellent tools for intervention studies aimed at defining the programming factors and have given invaluable insight into underlying programming mechanisms, for example, free wheel running was found to normalize the hyperinsulinemia, hyperglycemia, hyperleptinemia, hypertriglyceridemia and hypercholesterolemia observed in obese mothers at the end of lactation, whereas the effects on the young male offspring were a reduced fat accretion and plasma leptin two weeks after weaning^15^.

Insulin resistance is one of the earliest detrimental consequences of development within an obesogenic *in utero* environment and is observed in young adulthood, prior to changes in offspring body composition^16^. Molecular analysis has shown that the whole body insulin resistance is associated with changes in insulin signaling protein expression, especially in adipose tissue. At least some of these epigenetic changes such as reduced Insulin Receptor Substrate 1 (IRS-1) expression are cell autonomous and are retained in programmed primary pre-adipocytes that are differentiated *in vitro*^16^.

The first aim of this study was to identify maternal obesity programming factors through correlation analyses between offspring insulin resistance and maternal indices including body weight, fat mass and serum metabolites and hormones. The obese dams developed hyperinsulinemia, hyperleptinemia and hyperlipidemia as expected, however, insulin emerged as the only factor studied that correlated significantly with offspring hyperinsulinemia. Having identified maternal insulin as the programming factor, our second aim was to develop an intervention strategy to prove causality and to help define underlying mechanisms.

## Results

### Maternal serum insulin concentrations correlate with offspring serum insulin at 8 weeks of age.

**Figure 1:**
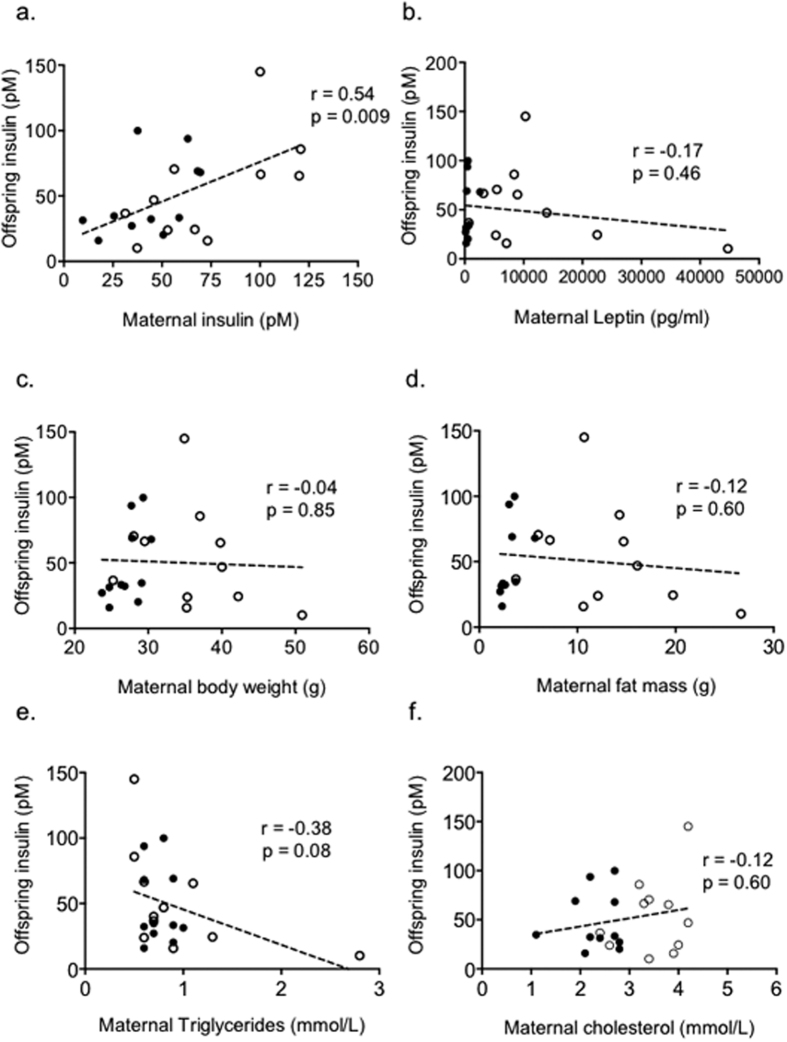
Male offspring insulin is correlated to (**a**) maternal fasting insulin, but not to (**b**) maternal leptin, (**c**) maternal body weight, (**d**) maternal fat mass, (**e**) maternal triglycerides or (**f**) maternal total cholesterol. Dams (n = 11 control and n = 11 obese) were mated, allowed to litter and litters from both groups standardised to 6 pups per litter (equal male to female ratio). At weaning, dams were weighed and body composition assessed by TD-NMR. Dams were then fasted overnight and killed the following morning, when blood was collected for serum analysis. Male offspring from the control and obese dam groups were maintained to 8 weeks of age, when they were killed and blood collected for serum analysis. Data was analysed by 2- tailed Spearman correlation (alpha = 0.05), and presented as filled circles for matched control dams and offspring and as open circles for matched obese dams and offspring.

Figure S1 depicts schematically the experimental protocol used. Maternal fasting serum insulin levels were found to correlate significantly with offspring fasting serum insulin at 8 weeks of age (Spearman r = 0.54, p = 0.009; Fig. 1a). There was however no correlation between maternal leptin levels (Fig. 1b), maternal body weight (Fig. 1c), or maternal fat mass (Fig. 1d) with offspring insulin levels. There was also no correlation between maternal triglycerides or cholesterol with offspring insulin levels (Fig. 1e,f). This suggests that maternal hyperinsulinemia is a key programming factor that mediates the effects of maternal over-nutrition on offspring insulin sensitivity.

### Exercise restores the obesity associated maternal insulin resistance without reducing body weight or total body fat.

**Figure 2:**
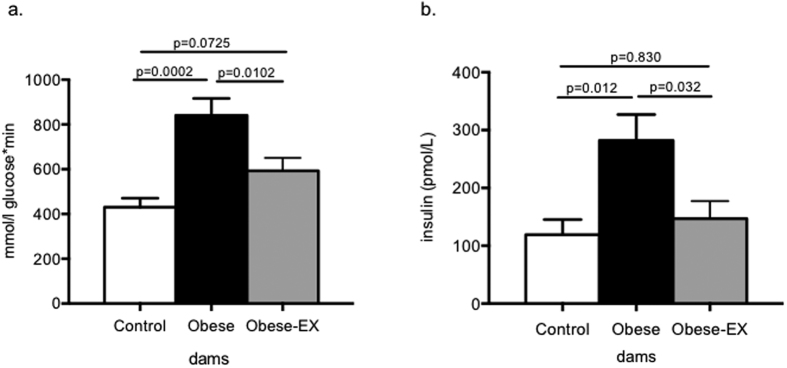
Glucose tolerance test at D18 of pregnancy and fed insulin concentrations at D19. On day 18 of gestation, control (white bars), obese (black bars) and obese-exercised (grey bars) dams were fasted for 4 hours, weighed and given an intraperitoneal dose of glucose (1 g/kg). Tail blood glucose was measured at time 0 (before the injection) and then again at timed intervals from the time of injection. Tail blood glucose measurements during the GTT were analyzed and presented as (**a**) area under the curve measurements calculated by summation of trapezoids and (**b**) fed serum insulin concentrations at E19 as measured by ELISA. Data are presented as means+/−SEM, n = 6 per group. The effects of obesity and exercise were analyzed by One-way ANOVA followed by Tukey’s multiple comparison test.

Having shown that maternal hyperinsulinemia is a potential factor driving the insulin resistance phenotype, we developed a regime of perigestational exercise to the mother aimed at improving her insulin sensitivity. The three experimental groups and the manner in which the exercise regime was implemented for the E19 gestational studies is described in the ***Methods*** and as depicted in Figure S2. At E18 of gestation, 4 hour fasting blood glucose was comparable in all 3 dam groups (Control 8.7 ± 0.8, Obese 8.6 ± 0.5, Obese-EX 8.3 ± 0.5 mmol/L). A glucose tolerance test was then performed and following an intraperitoneal bolus dose of 1 g/kg glucose, the glucose area under the curve (AUC) was significantly increased in the obese group compared to the other two groups (Fig. 2a). There was no difference in glucose AUC between the control group and the obese-exercised group. Glucose levels were significantly (p < 0.0001) elevated in the obese group compared to the control group at 15 mins post-glucose administration (Figure S3), and remained significantly higher than the control group (p < 0.0001) at 30 mins after glucose administration. At the 30 min time point, glucose levels in the obese group were also significantly higher (p = 0.0109) than the obese-exercised group (Figure S3). Fasting insulin concentrations were also significantly elevated in the obese group compared to the controls and were reduced to control values in the obese-exercised group (Fig. 2b). Maternal obesity also resulted in hyperleptinemia, however, levels remained high in the obese-exercised dams compared to the controls (Control 2171 ± 171, Obese 6064 ± 568, Obese-EX 5910 ± 388 pg/ml; Controls vs. Obese p < 0.0001, Controls vs. Obese-exercised p < 0.0001, comparisons by one-way ANOVA). This was consistent with the lack of effect of exercise intervention on body fat mass. Both at the beginning (day 1) and end of pregnancy (day 19), dams in both Obese groups were heavier than the Controls (Table 1). This difference in body weight was attributed to increased fat mass in the obese groups compared to the controls (Table 1). When comparing the obese group to the obese-exercised group, neither fat nor lean masses were altered by exercise, therefore on day 19 (at the end of the exercise intervention), there were no differences in body composition between these 2 groups. (Table 1).

### Exercise rescues the increased placental lipid deposition associated with maternal obesity

**Figure 3:**
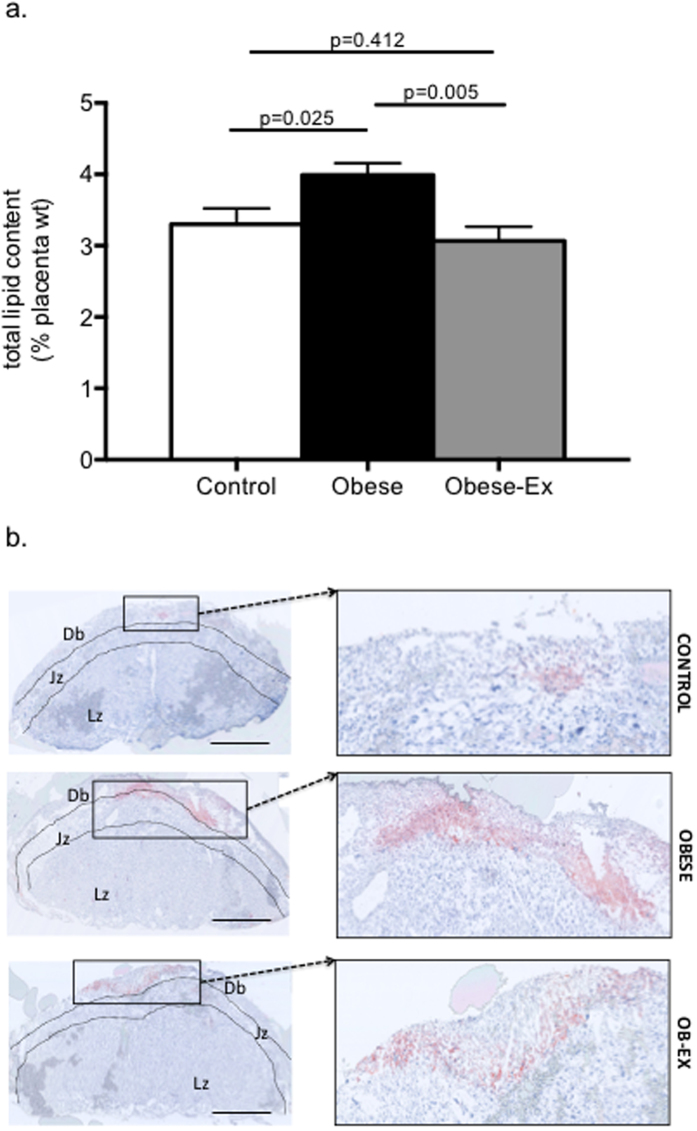
Lipid accumulation in the placenta (**a**) Total lipid content of placentae from control (white bars), obese (black bars) and obese-exercised (grey bars) dams were determined by Folch method and data presented as means+/−SEM, n = 6 per group. The effects of obesity and exercise were analyzed One-way ANOVA followed by Tukey’s multiple comparison test; (**b**) Oil red O-stained placentae sectioned from frozen tissue show increased lipid deposition within the decidual (Db)/junctional zone (Jz), but not in the labyrinthine zone (Lz) of obese placentae compared to controls, which is normalized by exercise, Scale bar = 100 μM.

Placental weights did not differ between the 3 groups (Control, 81.5 ± 1.2; Obese, 87.7 ± 3.8; Obese-Ex, 85.0 ± 2.3 mg). There were also no differences in placental weight when only male placentae were considered (Control, 83 ± 2.9; Obese, 87 ± 1.5; Obese-Ex, 85.7 ± 4.9 mg). Total lipid content as assessed by the Folch method was increased in obese placentae, but this was prevented by the maternal exercise intervention (Fig. 3a). Lipid content was also assessed by oil red-O staining of placenta in cross-section and quantification revealed that the area stained as a percentage of total placental area was significantly increased in obese placentae compared to controls, and brought down slightly but significantly by maternal exercise [Control, 0.52 ± 0.13; Obese, 1.47 ± 0.21; Obese-Ex, 0.82 ± 0.28 relative units; Control vs. Obese p = 0.009; Obese vs. Obese-Ex p = 0.045]. A similar pattern of lipid content was observed when only male placenta were analysed [Control, 0.74 ± 0.31; Obese, 1.82 ±0.14; Obese-Ex, 0.99 ± 0.45 relative units; Control vs, Obese p = 0.033; Obese vs. Obese-Ex p = 0.154]. The histology from this analysis also suggested that lipid primarily accumulated in the decidual/junctional zones (Fig. 3b).

### Maternal obesity is associated with placental hypoxia that is restored by exercise

**Figure 4:**
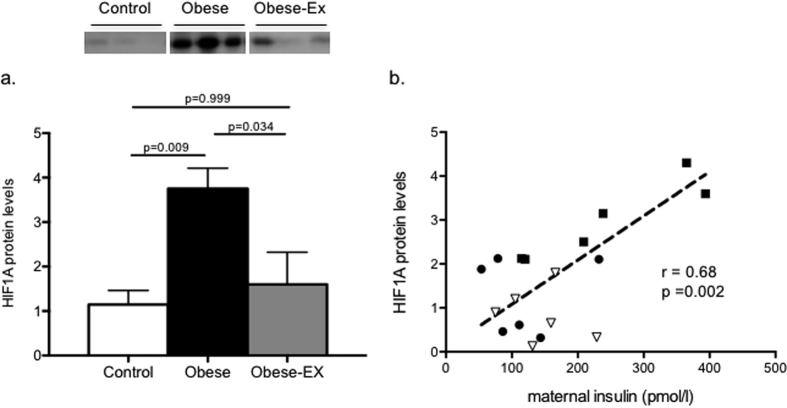
Placental HIF1A is upregulated by maternal obesity but attenuated by exercise. (**a**) HIF1A protein levels in placentae from control (white bars), obese (black bars) and obese-exercised (grey bars) dams, with representative blots shown above. Data are presented as means+/− SEM, n = 6 per group and the effects of obesity and exercise were analyzed by One-way ANOVA followed by Tukey’s multiple comparison test; (**b**) Pearson correlation between maternal insulin and placental HIF1A protein levels, 2-tailed p = 0.002 (alpha = 0.05) and r = 0.68, with data from matched groups displayed for controls as filled circles, obese as filled squares and obese-exercised as open triangles.

Western blotting showed placental hypoxia-inducible factor 1 alpha subunit (HIF1A) protein to be elevated by maternal obesity (Control vs. Obese p = 0.009), and restored to control levels with exercise (Obese vs. Obese-exercised p = 0.034) (Fig. 4a). This suggested that the placentae in the obese mice experienced hypoxia, which was ameliorated by exercise. A similar pattern was observed when only male placentae were considered, with obesity resulting in significantly elevated levels of HIF1A, and exercise intervention resulting in a trend to Control levels (Controls, 1.1 ± 0.424; Obese, 3.93 ± 0.691; Obese-Ex, 1.633 ± 0.994 relative units; Control vs. Obese p = 0.013; Obese vs. Obese-Ex p = 0.107). We then investigated if maternal insulin concentrations were associated with placental hypoxia, using HIF1A levels as a proxy, and found them to be significantly correlated (Fig. 4b, Pearson r = 0.68; p = 0.002), with a male-only analysis revealing a corresponding correlation between maternal insulin and male placental HIF1A levels (Pearson r = 0.711, p = 0.014). Transcript levels of *Hif1a* were however not affected either by maternal obesity or the exercise intervention (Control 1.0 ± 0.06, Obese 1.02 ± 0.15, Obese-Ex 0.94 ± 0.09; relative expression), with male only expression also being similarly unaffected (Control, 1.0 ± 0.07, Obese 0.92 ± 0.24; Obese-Ex, 0.97 ± 0.07), implying that the observed changes in HIF1A protein levels were not a result of increased transcription at this locus.

### Exercise rescues offspring hyperinsulinemia and adipose tissue insulin resistance

**Figure 5:**
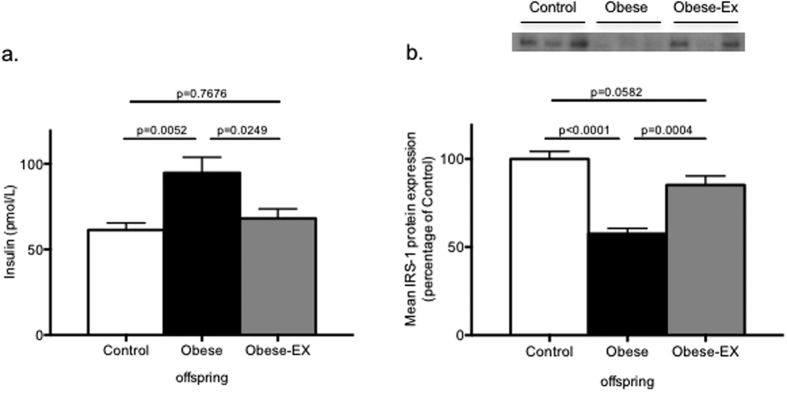
Insulin sensitivity in offspring of obese mothers is restored to control levels by maternal exercise. (**a**) Fasting serum insulin in male offspring at 8 weeks of age (**b**), Fasted IRS1 protein levels in epididymal adipose tissue of 8-week old male offspring of control (white bars), obese (black bars) and obese-exercised (grey bars) dams with representative blots shown above. Data are presented as means+/−SEM, n = 8 per group. The effects of obesity and exercise were analyzed One-way ANOVA followed by Tukey’s multiple comparison test.

In a previous study, we showed that offspring of obese mothers were hyperinsulinemic and that IRS1 protein levels were markedly suppressed in their epididymal adipose tissue^16^, a potential marker of insulin resistance in this fat depot at this early age. Thus, the effects of maternal exercise intervention were examined in the male offspring of all 3 maternal groups. Fasting serum insulin levels were measured in 8-week-old offspring and, consistent with our previous findings, the offspring of obese dams were hyperinsulinemic compared to the offspring of control dams (Fig. 5a; p = 0.0052), and importantly, exercise intervention in the obese dams prevented the development of this programmed hyperinsulinemia in the male offspring (Fig. 5a; Obese vs. Obese-exercised p = 0.0249). Furthermore, this was accompanied by a significant restoration of IRS-1 protein levels in their epididymal adipose tissue to almost control levels (Obese-exercised vs. Obese p = 0.0004; Fig. 5b), compared to a 50% loss in the offspring of obese dams (Obese vs. Controls p < 0.0001; Fig. 5b).

## Discussion

The aim of this study was to determine the key programming factor that mediates the effects of maternal obesity on offspring insulin sensitivity, with a view to identifying potential rational intervention strategies to halt the epidemic increases in metabolic diseases such as type-2 diabetes in both the developed and developing world. We have shown here that hyperinsulinema in obese mothers directly correlates with offspring hyperinsulinemia and insulin resistance. Furthermore, our maternal exercise intervention strategy, which improved maternal insulin sensitivity without altering maternal body weight, body composition, lipids levels or leptin levels strongly suggests that maternal insulin is an important contributing factor in the development of offspring insulin resistance and may mediate epigenetic effects. This observation is in contrast to other experimental approaches^15^ where voluntary free wheel running was adopted, when in addition to insulin levels being reduced, reductions in triglyceride, glucose and cholesterol levels were also observed. It is noted however that with voluntary free wheel running, it is likely the animals are running for much longer durations and distances than in our current study, which would explain why not all metabolic parameters were similarly affected here.

The pathways through which maternal insulin mediates its programming effects on the fetus are not known. Insulin is not thought to cross the placenta, however, the placenta expresses insulin receptors and is responsive to insulin with respect to lipid metabolism, since high levels of insulin have been shown to increase lipid accumulation^17^,^18^. Here, we observed increased lipid content in the placentae from obese dams, consistent with findings in obese human placentae where it has been localized in the villi stroma and syncytium^8^. In our model as well as in obesity-prone rats, lipid was found to be localized predominantly in the decidua^19^. One study in Fatp2 and Fatp4 knockout mice^20^ observed an increased lipid deposition in the decidual/junctional zones of the placentae, suggesting these fatty acid transporters are essential for the trafficking of lipids across the placenta. It is still unknown why lipids should accumulate at the decidual/junctional barrier, but it could be speculated that this might, structurally at least, interfere with angiogenesis and the capacity of maternal vessels to invade the labyrinthine trophoblast and therefore impair the exchange of nutrients and gases between mother and fetus. The observation that maternal exercise prevented an increase in placental lipid deposition in parallel with a reduction in maternal insulin levels suggests that hyperinsulinemia and therefore increased insulin action on the placenta may contribute to the increased placental lipid deposition. This proposed action of insulin is supported by recent studies^21^ which investigated the impact of maternal obesity on lipid metabolism genes in human placenta. Here, the authors observed a two-fold increase in protein abundance of a master regulator of triglyceride hydrolysis, CGI-58, in placentae of obese women compared to lean placentae, which positively correlated with maternal insulin levels. In addition, they showed that *in vitro* treatment of cultured trophoblast cells with insulin induced the expression of CGI-58.

Maternal obesity was also associated with increased placental HIF1A protein expression. HIF1A is an important molecular sensor of oxygen levels in the placenta and is induced when oxygen tension is low^22^. Hence, a rise in HIF1A protein abundance supports the hypothesis that obesity during pregnancy results in placental hypoxia. Hypoxia has been shown to directly increase lipid accumulation in human trophoblasts^23^, therefore it may also contribute to the increased placental lipid accumulation. Other factors that may contribute to a hypoxic state include inflammation^24^,^25^ and mitochondrial dysfunction^26^ which are observed preferentially in female placentae, while other factors such as oxidative stress^27^ and ER stress^28^ appear to be sex-independent.

While there are differences in mouse and human gestation and placental structure, there are many common functional characteristics between the two species, particularly nutrient transfer, both in normal and pathological situations^29^. Structurally, feeding an obesogenic diet during pregnancy has been shown to result in a reduced fetal capillary volume^30^ which is associated with impaired oxygen delivery^31^. This supports the idea of a hypoxia-mediated response to maternal obesity in the placenta. Further support for this comes from the observation that placental HIF1A protein levels are brought back down to control levels as a consequence of maternal exercise.

There are several mechanistic pathways via which maternal insulin may be inducing placento-fetal hypoxia. Firstly, insulin is a nitric oxide (NO)-dependent vasodilator^32^ and endothelial insulin-mediated vasodilatation has been shown to be mediated by endothelial nitric oxide synthase (eNOS)^33^. Human studies and animal models strongly link insulin resistance with decreased NO bioavailability: In humans, NO-mediated insulin-induced vasodilatation is impaired in insulin-resistant states^34^; and NO bioavailability is known to be reduced both in animal models of obesity and diabetes^35^ and in obese and diabetic humans^36^. Also, a deletion in NOS3, one of the three enzyme isoforms of nitric oxide, causes insulin resistance, glucose intolerance, hyperlipidaemia and hypertension^37^. We hypothesize therefore that maternal hyperinsulinemia/insulin resistance may negatively affect the amount of available NO, thereby impairing uteroplacental vasodilation, leading to decreased placental perfusion and ultimately resulting in placental-fetal hypoxia^38^.

Maternal hyperinsulinaemia may also directly affect placental HIF1A levels. The effects of maternal obesity and the exercise intervention on HIF1A protein levels ensued without any change in *Hif1a* mRNA suggesting that they might be mediated through a post-transcriptional mechanism.

A central finding of our study was that maternal insulin levels were the strongest predictor of male offspring insulin resistance and that this occurred independently of maternal or offspring^16^ weight. Designing an intervention that specifically improved maternal insulin sensitivity, without impacting on other potential factors such as maternal weight, body composition, lipid or leptin levels, allowed us to demonstrate a causal relationship between maternal insulin and offspring insulin resistance. The improvement in maternal insulin sensitivity prevented the development of insulin resistance in the offspring and a loss of IRS-1 protein in their epididymal adipose tissue, a depot which has been documented as representative of human visceral fat^39^, a depot associated with insulin resistance in humans. We also previously showed that primary preadipocytes isolated from this depot and then differentiated *in-vitro*, maintain this loss of IRS-1 protein and are thus programmed epigenetically by maternal obesity in a cell-autonomous fashion^16^. Our studies have also shown that the mature adipocytes in this depot are larger in size and demonstrate an inflammation phenotype^40^. These observations are highly consistent with the work of others^15^ and underlines that the detrimental metabolic effects of perigestational obesity on both the mother and her offspring may be rescued by moderate exercise.

In conclusion, we have shown that maternal insulin is a key programming factor mediating the effects of maternal diet-induced obesity on offspring insulin sensitivity. As well as providing mechanistic insight, our findings have important translational implications as they propose that in situations of maternal obesity, it may be better to focus on interventions that improve metabolic fitness rather than control maternal body weight. This is important in light of recent human studies that have shown that it is possible to modulate behavior in obese pregnant women^14^,^41^, even though it will be many years before evidence for the potential long-term effects of these interventions are observed in the offspring. Our findings suggest that providing women with the tools and information on the benefits of incorporating an active lifestyle prior to and during pregnancy could have huge potential long-term benefits in preventing transmission of obesity from mother to child.

## Methods

### Animals and Diets

All protocols were approved by the animal welfare ethical review process of the University of Cambridge and carried out under licence from the U.K. Animals (Scientific Procedures) Act 1986. Female C57BL/6 J mice were randomly assigned to one of two diets fed *ad libitum* throughout, i.e. either a standard chow RM1 diet (approx. 7% simple sugars, 3% fat, 50% polysaccharide, 15% protein [w/w], 10.74 kJ/g) or a semi-synthetic energy-rich highly palatable obesogenic diet (approx. 10% simple sugars, 20% animal lard, 28% polysaccharide, 23% protein [w/w], 28.43 kJ/g) supplemented with sweetened condensed milk (Nestle, UK) (approx. 16% fat, 33% simple sugars, 15% protein, 13.7 kJ/g) which was fortified with mineral and vitamin mix AIN93G. Both diets were purchased from Special Dietary Services (Witham, UK). After three weeks on the respective diets, females were mated with chow-fed males for their first pregnancy. Dams remained on their respective diets throughout pregnancy and lactation and the first litter was culled after weaning. The first pregnancy ensured fertility and nurturing in the experimental mice. One week after weaning, body composition in the female dams was analyzed by Time Domain Nuclear Magnetic Resonance (TDNMR). From previous studies, we had established that for a consistent phenotype to be observed in the offspring of obese dams, the obese dams’ body composition should be around 30–35% fat and that body weight should not exceed 35 g prior to mating for second pregnancy. The body composition of the Control dams that had been fed the standard chow throughout life was also measured and body fat in this instance was not permitted to exceed 10–12% prior to mating for second pregnancy.

### Maternal - offspring correlations

For the correlation studies, dams (n = 11 control and n = 11 obese biological replicates, where each dam mother is defined as the statistical unit) were set up as above and mated for a second pregnancy, then allowed to litter. At 48 hours post-partum, litters from all dams were standardized to 6 pups per dam (equal male:female ratio). At weaning, dams were fasted overnight and the next day they were killed and blood collected for serum analysis. Weaned offspring were maintained on the standard chow from weaning to 8 weeks of age. At 8 weeks of age, 1 male offspring from each litter was randomly selected for study, fasted overnight and killed the next day. Blood was then collected for serum analysis.

### Gestational studies

A separate cohort of n = 6 control and n = 12 obese dams were used in the pregnancy studies. As before, the dams’ body composition was monitored before mating. Half of the obese group (n = 6) that was not exercised were placed on a static treadmill to control for environmental differences while and the other half (n = 6) were entered into a regime of physical exercise as described below. Timed mating for all 3 groups of dams (n = 6 biological replicates, where each dam mother is defined as the statistical unit) were conducted using the same set of male studs and day 1 of pregnancy was defined by the appearance of a copulatory plug. Dams were maintained on their respective control or obesogenic diets (obese and obese-exercised groups) throughout both pregnancies and lactation periods. Weekly weights of the dams were recorded and body composition was measured at D1 and D19 of pregnancy.

#### Exercise intervention

We used an exercise strategy rather than a pharmacological approach as it is likely to be more translatable^41^, with recent studies demonstrating that exercise during obese pregnancy can improve insulin sensitivity and reduce the prevalence of gestational diabetes^42^. Although Standford *et al*.^43^ showed recently that exercising pregnant mice that were fed a high fat diet for 2 weeks prior to mating led to an improvement in metabolic health of the offspring, it is important to point out the major differences between the two models. In their model, mice were concurrently fed a high fat diet while being exercised 2 weeks before mating, such that the dams’ body weight, fat mass and glucose tolerance in that study was not at any time different to their controls. In contrast, our model is one of perigestational maternal obesity, as the dams are maintained on an obesogenic diet for 6 weeks prior to gestation and the obese dams accrue three times the fat mass of a lean control dam even before entering gestation, thus modelling more accurately the human obesity problem. Taking this model forward, we imposed a regime of daily exercise on the obese dams a week prior to gestation and up to 17 days during gestation, which led to a significant rescue of the key factor found to correlate with offspring hyperinsulinemia and insulin resistance, i.e. maternal hyperinsulinemia.

From one week prior to mating for the second pregnancy, the obese dams under exercise intervention protocol (n = 6) were trained to run 20 minutes daily on a treadmill, starting at 5 m/min on the first day and with daily incremental increases in speed until they were running at a final speed of 12.5 m/min on day 5. Dams were then rested for 2 days during the weekend when they were time-mated and day 1 of pregnancy was defined by the appearance of a copulatory plug. Exercise was then maintained at the final running speed and following a routine of 5 days of daily exercise (weekdays) followed by 2 days of rest (weekends), until day 17. The duration of running at the top speed of 12.5 m/min was reduced in the final 3 days to adapt to the physical constraints of pregnancy.

#### Maternal glucose tolerance

On day 18 of gestation, mice dams from all three groups (Control, Obese and Obese-exercised) were fasted for 4 hours between 0730 and 1130, and blood drawn from the tail for basal glucose measurements (AlphaTRAK, Abbot Logistics B.V., Netherlands). Dams were then injected intraperitoneally with 1 g/kg glucose and further tail blood glucose measurements were made at timed intervals. AUC was calculated by summation of trapezoids (Prism, GraphPad, La Jolla, USA).

#### Maternal blood and tissue collection

Dams were re-fed after the GTT and the following morning (day 19 gestation) they were killed by rising CO_2_ concentration. Blood was sampled by cardiac puncture and serum obtained and stored at −80 °C for further analysis.

#### Placental analyses

Whole placentae were collected from each fetus and assigned to various experiments as shown below. Placentae of around median weight of the whole litter were selected for a) Oil red O analysis; b) total lipid content and c) molecular analysis (protein, RNA). 3 male and 3 female whole placentae in all three groups were used to rule out any contributions arising from sex differences. Fetuses were sexed by visual inspection for testes, and yolk sacs DNA was subjected to PCR for *Sry*, where an amplification of *Sry* gene confirmed the fetus and associated placenta as male.

For oil-red O staining, frozen whole placenta sections of 7 μm thickness were stained with Oil Red O (Sigma-Aldrich, UK) and counterstained with Mayer’s Haematoxylin (Sigma, St. Louis, MO, US) to detect lipid accumulation within the various structures. Images were captured on an inverted light microscope (Olympus BX41, Olympus UK Ltd, Southend-on-Sea, UK). Single blinded analysis was carried out using Adobe Photoshop Elements (Adobe Systems Inc., San Jose, USA) on two fields of view from two sections per sample (n = 7 per group). Lipid droplets (red) were selected using the magic wand tool and quantified as a percentage of positively stained pixels in the field of view. Total lipid was quantified by a modified Folch method^44^.

For gene expression analyses, total RNA was isolated from placentae using DirectZol (Zymogen, Cambridge Biosciences, UK). cDNA was generated using a High-Capacity cDNA Reverse Transcription Kit (Applied Biosystems, UK) and quantitative real time PCR (qPCR) performed on a StepOnePlus™ Real-Time PCR System (Applied Biosystems), using 400 nM primer concentrations, (Sigma-Aldrich, UK), 1× SYBR Green master mix (Applied Biosystems, UK) and a 1:10 dilution of sample cDNA. Quantification of mRNA levels was performed relative to a standard curve which was generated using dilution series of the pooled cDNA samples. mRNA expression of *Hif1a* was normalized to the geometric mean of selected reference genes *B-Act, Ppia, Pabp1* and *Hprt*. Primer sequences used in the PCR analysis are listed in Supplementary Table 1.

For protein analyses, one whole placentae from each litter (close to the median weight) was processed for western blotting as further described below.

### Offspring studies

A third cohort of control, obese and obese-exercised dams (n = 8 dams per group; biological replicates, where each dam mother is defined as the statistical unit) were allowed to litter and litters from all 3 groups were standardized to 6 pups (3 males and 3 females) at 48 hours post-partum. Offspring from all 3 groups were then weaned onto standard chow until 8 weeks of age. A single male offspring from each litter was selected at random at 8 weeks of age, fasted overnight and killed the following morning. Blood was collected and allowed to clot. Serum was then obtained for the analysis of fasting insulin concentrations and epididymal adipose tissue was collected for western blotting analysis of insulin receptor substrate-1 (IRS-1) protein levels, which allowed us to investigate the effects of maternal exercise on these offspring parameters. 100 mg of epididymal adipose tissue was processed for western blotting.

### Western blotting

Tissue was disrupted by mechanical homogenization (TissueRuptor, Qiagen, Manchester, UK) in lysis buffer (50 mmol/l HEPES, pH 8; 150 mmol/l sodium chloride; 1% Triton X100; 1 mmol/l sodium orthovanadate; 30 mmol/l sodium fluoride; 10 mmol/l sodium pyrophosphate; 10 mmol/l EDTA and a protease inhibitor cocktail (set III, Calbiochem Novabiochem Biosciences, Nottingham, UK). Nuclear material was pelleted by centrifugation at 12,000 g and the clear supernatant removed to a fresh tube. Total protein concentrations in the cleared lysate were measured using a copper/bicinchoninic assay (Sigma-Aldrich, UK). Samples from all 3 groups (20 μg total protein) were subjected to SDS-PAGE and transferred onto a PVDF membrane (Immobilon-P, Millipore, Billerica, MA, USA). To ensure signal linearity, 20 μg and 10 μg of a pooled sample (from all 3 groups) were loaded in each gel. A 2:1 ratio of optical density in the bands of interest confirmed that the analyses were within dynamic range. Following a blocking step in 5% Bovine serum albumin (Sigma, UK), 1× Tris-buffered saline (TBS), 0.1% Tween 20, the PVDF membrane was incubated overnight with the primary antibodies of interest. This was followed by washing with 1× TBS, 0.1% Tween 2, after which membranes were incubated with horseradish peroxidase-conjugated anti-rabbit or anti-mouse antibody (Jackson ImmunoResearch, Stratech, UK). Antibody binding was detected using Super Signal West Pico Chemiluminescent substrate (Thermo Scientific, UK) and the chemiluminescent bands were quantified directly with ImageQuant LAS4000 (GE Healthcare Life Sciences, UK). The following antibodies were used: IRS-1 (Upstate Biotechnology, Millipore, Lake Placid, USA), HIF1A (R&D systems, UK). Each immunoblot experiment was performed three times for a total of 3 technical replicates per antibody.

### Serum analysis

Maternal and male offspring serum insulin concentrations were measured using an Ultrasensitive Mouse Insulin ELISA kit (Crystal Chem, USA). Maternal leptin concentrations were measured using a Mouse Leptin ELISA kit (Crystal Chem, USA). Samples were assayed in duplicate and intra and inter-assay coefficients of variation of less than 10.0% was considered acceptable for both assays.

### Statistical Analyses

Calculations for sample size were obtained by factoring in a p-value of 5% significance level, 90% power and the standard deviation of the difference between the groups (StatMate, GraphPad, La Jolla, USA). The accepted protocol in programming studies is that only one offspring of each litter is represented as the *N* and the dam is the statistical unit. Two-sided tests were performed for all statistical evaluations (Prism, GraphPad). One-way ANOVAs, followed by Tukey’s correction for multiple comparisons were used to test differences between the three dam groups to first identify the significant effects of obesity and the effects of exercise intervention on obesity. Changes between day 1 and day 19 of pregnancy within the same groups were analyzed by paired t-tests. Placental data were averaged for each litter before calculation of mean values for each gestational age.

## Additional Information

**How to cite this article:** Fernandez-Twinn, D. S. *et al*. Exercise rescues obese mothers’ insulin sensitivity, placental hypoxia and male offspring insulin sensitivity. *Sci. Rep.* **7**, 44650; doi: 10.1038/srep44650 (2017).

**Publisher's note:** Springer Nature remains neutral with regard to jurisdictional claims in published maps and institutional affiliations.

## Supplementary Material

Supplementary Information

## Figures and Tables

**Table 1:** Body weight and body composition of Control, Obese and Obese-exercised dams at D1 and D19 of gestation.

		Control	Obese	Obese-Ex	Control vs. Obese	Control vs. Obese-Ex	Obese vs. Obese-Ex
					p-value of multiple comparisons		
*Body weight*							
	Day 1	27.56 ± 0.71	36.39 ± 1.19	33.12 ± 1.36	0.0006	0.0130	0.2123
	Day 19	43.38 ± 0.71	50.12 ± 2.44	47.38 ± 1.52	0.0383	0.2558	0.5050
*Body composition*							
Fat mass	Day 1	12.05 ± 0.58	37.17 ± 2.80	32.14 ± 3.21	0.0001	0.0017	0.2617
	Day 19	12.81 ± 0.50	31.16 ± 1.58	28.57 ± 0.98	<0.0001	<0.0001	0.1132
Lean mass	Day 1	70.80 ± 1.28	54.83 ± 1.69	59.17 ± 2.79	0.0003	0.0042	0.3181
	Day 19	80.11 ± 0.81	63.29 ± 1.21	59.42 ± 4.21	0.0016	0.0003	0.5532

Data are expressed as means ± SEM; n = 6 dams per group. The effects of obesity and exercise were analyzed by One-way ANOVA followed by Tukey’s multiple comparison test and significant effects of multiple comparisons are reported.
