# Supplementary material for: Exercise rescues obese mothers’ insulin sensitivity, placental hypoxia and male offspring insulin sensitivity
Source: Sci Rep. 2017 Mar 14;7:44650. doi: 10.1038/srep44650 (PMC5349590; doi:10.1038/srep44650)
Supplement: Supplementary Information [file srep44650-s1.pdf]

Supplementary Information

Title: Exercise rescues obese mothers' insulin sensitivity, placental hypoxia and male offspring insulin sensitivity

Authors and affiliations:

Denise S. Fernandez-Twinn[1*], Geraldine Gascoin[2], Barbara Musial[3], Sarah Carr[1], Daniella Duque-Guimaraes[1], Heather L. Blackmore[1], Maria Z. Alfaradhi[1], Elena Loche[1], Amanda N. Sferruzzi-Perri[3], Abigail L. Fowden[3], Susan E. Ozanne[1]

1 University of Cambridge Metabolic Research Laboratories and MRC Metabolic Diseases Unit, Wellcome Trust-MRC Institute of Metabolic Science, Cambridge, UK

2 Department of Neonatal Medicine, University Hospital of Angers, Angers, France

3 Department of Physiology Development & Neuroscience, University of Cambridge, UK

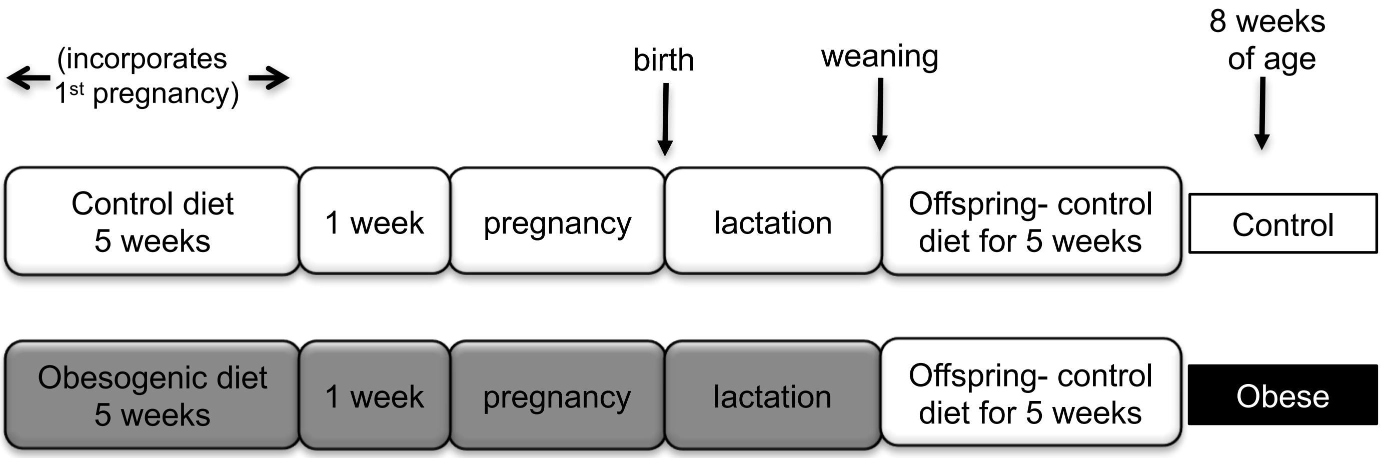

Figure S1: Experimental protocol to generate control and obese dams and their offspring. Blood for serum analysis was collected from dams at weaning and from 8 week old offspring (n=8 per group).

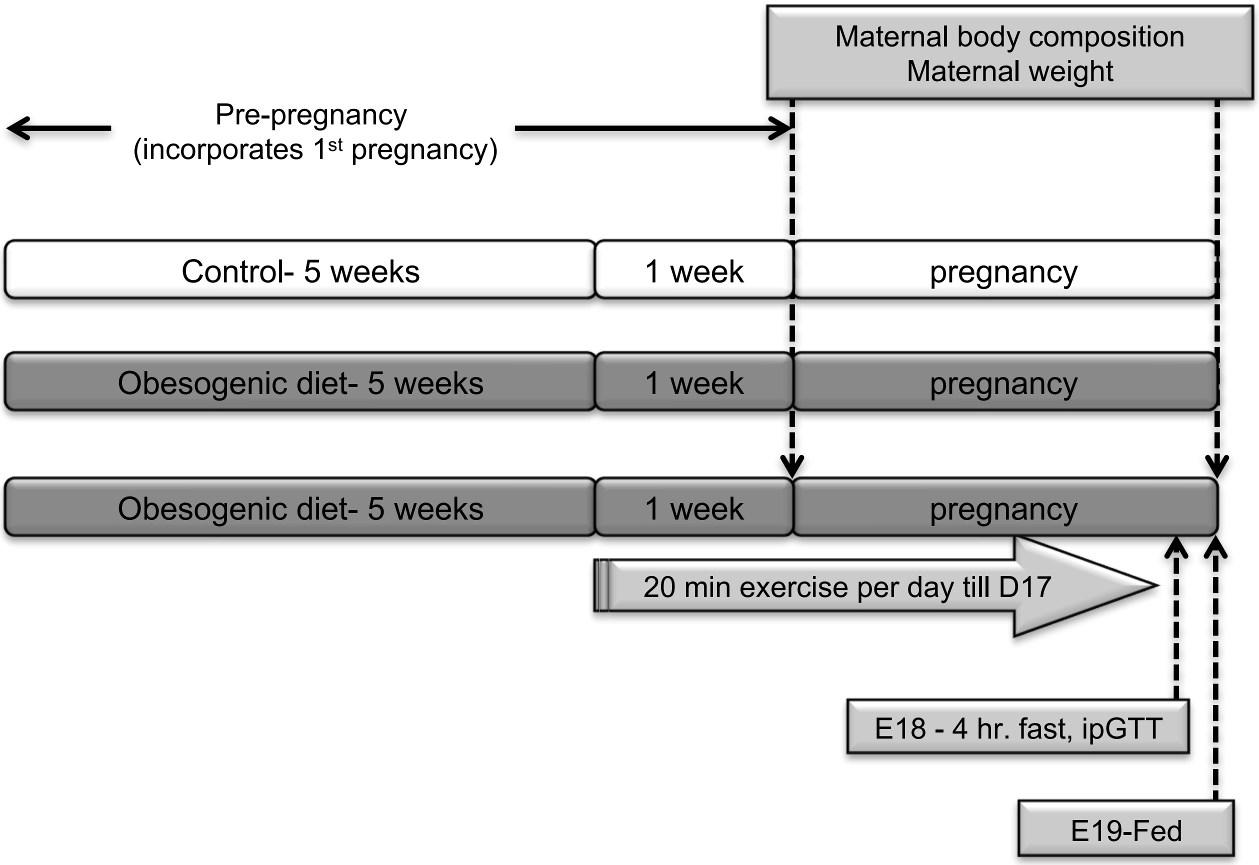

Figure S2: Schematic of study design incorporating an exercise regime for obese females prior to and during pregnancy (n=6 dams per group).

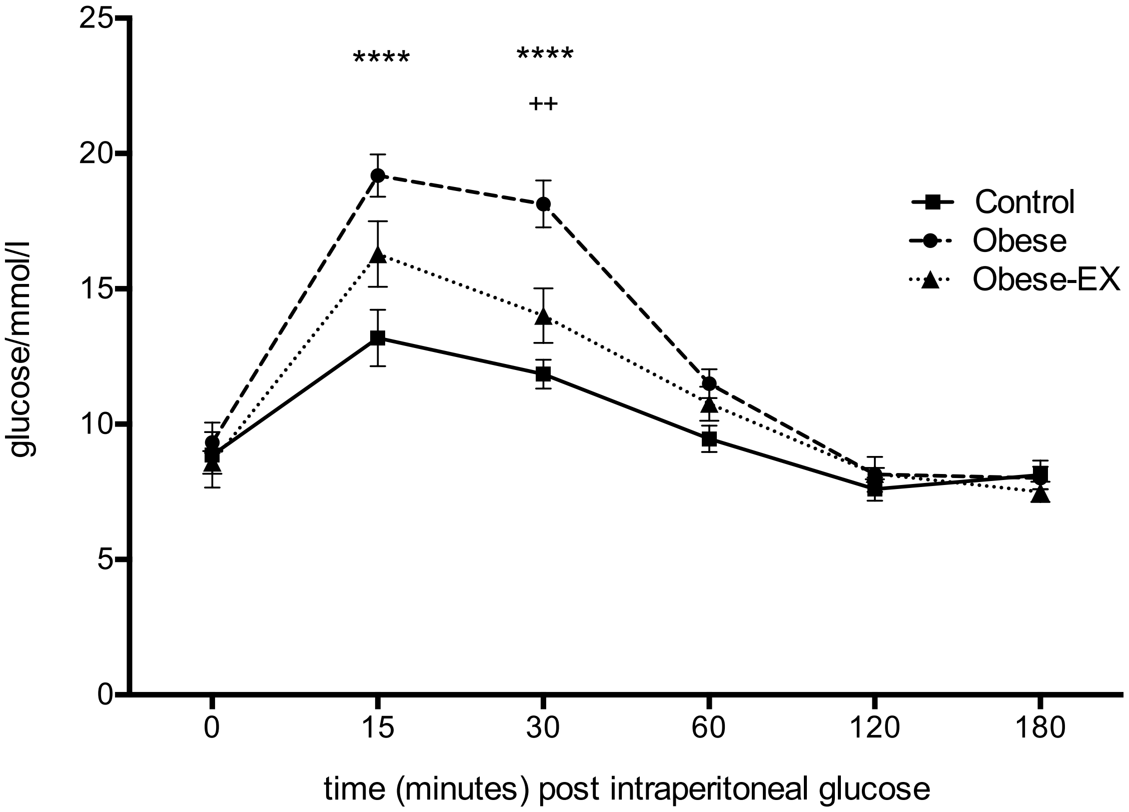

Figure S3 : Glucose tolerance test at D18 of pregnancy. On day 18 of gestation, dams were fasted for 4 hours, weighed and given an intraperitoneal dose of glucose (1g/kg). Tail blood glucose was measured at time 0 (before the injection) and then again at timed intervals from the time of injection. Tail blood glucose measurements throughout the GTT. Glucose levels (Controls- squares, Obese- circles, Obese-EX- triangles) are presented as means +/- SEM, n=6 dams per group and analyzed by two-way repeated measures ANOVA with Bonferroni multiple comparisons test. **** $p<0.0001$ Control vs. Obese; $^{++}p=0.0109$ Obese-EX vs. Obese.

Supplementary Table 1: Forward and reverse primer sequences

**Primer**	**Forward 5’-3’**	**Reverse 5’-3’**
**Sry**	TCATGAGACTGCCAACCACAG	CATGACCACCACCACCACCAA
**Hif1a**	GCGAGAACGAGAAGAAAAAGATGA	GGGGAAGTGGCAACTGATGA
**Hprt**	GCTCGAGATGTCATGAAGGAGAT	AAAGAACTTATAGCCCCCCTTGA
**B-Actin**	TTCAACACCCCAGCCATGTA	TGTGGTACGACCAGAGGCATAC
**Ppia**	AGGGTGGTGACTTTACACGC	GATGCCAGGACCTGTATGCT
**Pabp1**	ATGTTGGGTGAACGGCTGTTTCCT	GAGCGGAGAGACTCTGGAGACTCG
